# Metabolic changes in acute cerebral infarction: Findings from proton magnetic resonance spectroscopic imaging

**DOI:** 10.3892/etm.2013.1418

**Published:** 2013-11-19

**Authors:** AI-QIN LIN, JI-XIN SHOU, XUE-YUAN LI, LIN MA, XIAO-HAN ZHU

**Affiliations:** 1 Nursing School, Zhengzhou Railway Vocational and Technical College, Zhengzhou, Henan 450052, P.R. China; 2 Department of Neurology, Fifth Affiliated Hospital, Zhengzhou University, Zhengzhou, Henan 450052, P.R. China

**Keywords:** acute cerebral infarction, magnetic resonance spectroscopy, diagnosis, N-acetylaspartate, lactate

## Abstract

The purpose of this study was to investigate the clinical role of proton magnetic resonance spectroscopy (^1^H-MRS) in the diagnosis of acute cerebral infarction. Using databases available at the Fifth Affiliated Hospital of Zhengzhou University (Zhengzhou, China), the medical records of 47 patients with acute cerebral infarction treated between April 2010 and March 2012 were retrospectively reviewed. The patients underwent routine magnetic resonance imaging (MRI), diffusion-weighted imaging (DWI) and multiple-voxel ^1^H-MRS examination within 12 h after the onset of stroke. The patients then received normal medical treatment for 2 weeks and underwent follow-up ^1^H-MRS examination at 1–2 months after stroke. The concentrations of the main metabolites [N-acetylaspartic acid (NAA), creatine (Cr), choline (Cho) and lactate (Lac)] in the infarct center, the infarction border region and the contralateral brain areas (control) were analyzed. The 47 patients experienced changes in NAA, Cho and Lac levels at different stages after stroke. In the infarction center, the NAA/Cr and NAA/Cho ratios decreased, while the Lac/Cr ratio increased within 12 h compared with those in the contralateral side. Within 6–12 h after stroke, the Lac/Cr ratio increased and the NAA/Cho ratio decreased compared with those <6 h after stroke. During the 1–2 months post-stroke, significant reductions in the NAA/Cr, NAA/Cho, Cho/Cr and Lac/Cr ratios were observed in the infarction center. In the infarction border region, the Lac/Cr ratio increased significantly at 12 h and decreased during the 1–2 months after stroke. The NAA/Cr, NAA/Cho and Cho/Cr ratios were significantly increased in the infarction border regions of patients who received thrombolytic therapy for 1–2 months compared with those in patients who did not undergo thrombolysis. Our results highlight the usefulness of ^1^H-MRS-based metabolomics as a feasible and efficient prognostic tool for assessing the treatment effect of acute cerebral infarction.

## Introduction

Cerebral infarction, also known as ischemic stroke, seriously impairs the health of individuals (1). It is ischemic necrosis of an area of the brain due to blocked blood vessels. The disease is one of the significant causes of mortality and disability in older individuals (2). Ischemia of the brain tissue is in a reversible stage 6 h after stroke. Thrombolytic treatment is able to restore blood flow perfusion in this time, to reduce neurological damage and save the ischemic penumbra (3). Routine computed tomography (CT) and magnetic resonance imaging (MRI) play important roles in the diagnosis of cerebral infarction, and are used to elucidate the pathophysiological characteristics of brain infarcts and define the extent of tissue damage (4), but are less sensitive for diagnosing acute cerebral infarction. Clinically, CT and routine MRI are commonly used to assess the swelling of ischemic nerve cells and changes of the tissue structure caused by necrosis to determine the occurrence of cerebral infarction. Tissue changes often occur during the first few hours after a stroke and serious metabolic disorders of neurocytes may be observed (5,6). When signal changes are identified by CT, cells often have already undergone ischemic necrosis (6,7).

As a result of the development of proton magnetic resonance spectroscopy (^1^H-MRS), it is now possible to obtain further information concerning the spatial distribution of a spectroscopically visible metabolite in normal and ischemic human brain (8). ^1^H-MRS is able to monitor the metabolic changes that reflect the state of substance and energy metabolism in nerve cells by determining levels of certain metabolites in brain tissue. A recent study has observed that certain neurometabolic changes occur following stroke, but there is a lack of ^1^H-MRS studies of patients with acute cerebral infarction (9).

^1^H-MRS may be used to obtain noninvasive and *in vivo* determinations of the concentration of numerous intracellular metabolites and to analyze the tissue metabolic state based on the levels of these metabolites. N-acetylaspartic acid (NAA) is a marker of neuronal density that is synthesized by neuronal mitochondria and then distributed in the neuronal cell bodies; it also directly reflects the functional state of neurons. The inhibition of neuronal metabolism causes a significant reduction in the level of NAA in cerebral ischemia, and depletion of NAA when the infarction is irreversible (10). Thrombolytic therapy within 6 h after the stroke is of significant importance for restoring neurological function, and improving the survival and quality of life of the patient (5), thus early diagnosis and treatment are of great significance.

The purpose of this study was to analyze the advantages of spectroscopic imaging in acute cerebral infarction. Spectroscopic imaging permits evaluation of the entire extent of a pathological lesion and comparison with contralateral brain tissue. Of particular interest is the extent to which such spectroscopic changes correspond to ‘conventional’ imaging findings, and whether spectroscopic data allow allocation of different regions within large infarcts to different extents of brain alterations.

## Materials and methods

### Clinicopathological data

A retrospective analysis of the medical records of 47 patients (27 males and 20 females; aged 37–72 years; mean age, 57.0±8.9 years) with cerebral infarction, who were admitted to the Department of Neurology, Fifth Affiliated Hospital of Zhengzhou University (Zhengzhou, China) from April 2010 to March 2012 was performed. The time of stroke onset was within 12 h of symptom occurrence. Clinical symptoms included headache, dizziness, hemidysesthesia, hemiplegia, slurred speech and vision disturbance. The patients had MRI examinations using an Achieva 3.0T scanner, which has pre-^1^H-MRS detection analysis software and a standard head coil (Philips, Amsterdam, The Netherlands) and ^1^H-MRS examination. Once the diagnosis of cerebral infarction had been made, according to Chinese treatment guidelines of the acute ischemic stroke 2010 (11), thrombolytic therapy was administered to patients with indications for thrombolytic treatment and consent of their families was obtained as soon as possible, while the remaining patients underwent regular medical treatments (including anticoagulant, improvement of blood circulation, antiplatelet and cerebral protection). There were 43 patients reviewed who underwent ^1^H-MRS within 1–2 months after stroke.

### ^1^H-MRS detection and analysis procedures

The patients were managed by regular emergency treatment (patients were examined to assure stable vital signs, including blood pressure, which was controlled below 180/110 mmHg) following admission, and underwent head CT scanning to exclude intracranial hemorrhage and large area lesions as promptly as possible. The patients who met the inclusion criteria underwent ^1^H-MRS when their vital signs were stable and as soon as they could be removed from the electrocardiogram (ECG) and were able to withstand ~30 min of MRI inspection. The inclusion criteria were as follows: sudden headache, dizziness, hemidysesthesia, hemiplegia, slurred speech and vision disturbance. The patients were examined with CT to exclude intracranial hemorrhage and then examined via MRI to confirm the occurrence of infarction. The examinations were completed within 12 h. The patients were attended to during MRI examinations by neurologists.

All patients underwent routine MRI and ^1^H-MRS scans. Routine MRI sequences included axial, sagittal T_1_-weighted imaging (T_1_WI), T_2_WI and diffusion-weighted imaging (DWI); layer thickness was 5 mm in order to determine the pathological conditions of cerebral tissue. The sequence of axial T_2_WI or DWI identified the infarct lesions and positions for ^1^H-MRS. The volume of a single voxel was 2×1×1 cm. Water signals were suppressed by the chemical shift saturation method. The sequence of S_2_DSI-144 was applied to collect spectrum, imaging parameters were as follows: repetition time (TR) was 2,000 msec, echo time (TE) was 144 msec and number of excitations (NEX) was 16 times. FuncTool Spectroscopy-2D Brain analysis software (Philips Signa Workstation 4.0, Philips, Asterdam, The Netherlands) was used to analyze and calculate the integral peak area of the corresponding chemical shift of NAA, total creatine (creatine + phosphocreatine) (Cr), choline compounds (Cho), and lactate (Lac) automatically, which provided a relative quantitative value of the concentration of these compounds. The values of the NAA/Cr, Cho/Cr, NAA/Cho, and Lac/Cr ratios were calculated and analyzed.

Two senior experienced MRI diagnostic and operating physicians, with 20 years of combined experience in imaging analysis, performed independent diagnoses and made the final decisions. Discussions were used to determine the decision when conflicting opinions arose.

### Statistical analysis

The measurement data are expressed as mean ± standard deviation. The differences between the identical voxels located in the two different brain hemispheres of the group of patients and the same voxels tested at different times were analyzed using the paired Student’s t-test. Comparisons of two independent samples were performed by using the Student’s t-test. All statistical analyses were performed using SPSS 13.0 software (SPSS, Inc., Chicago, IL, USA). P<0.05 was considered to indicate a statistically significant result.

## Results

### Routine MRI findings

Seventy-two lesions (61 supratentorial lesions and 11 infratentorial lesions) were observed in 47 patients. On T_2_WI there was a slightly increased signal focus in 58 lesions with fuzzy boundaries and uniform signals in the other 14 lesions. Sixty-nine lesions showed a high signal on fluid attenuated inversion recovery (FLAIR) and the boundaries were clearer than those on T_2_WI. Three lesions showed a uniform signal. All 72 lesions showed a clearly highlighted signal in DWI.

### ^1^H-MRS findings in the acute phase

**Figure 1:**
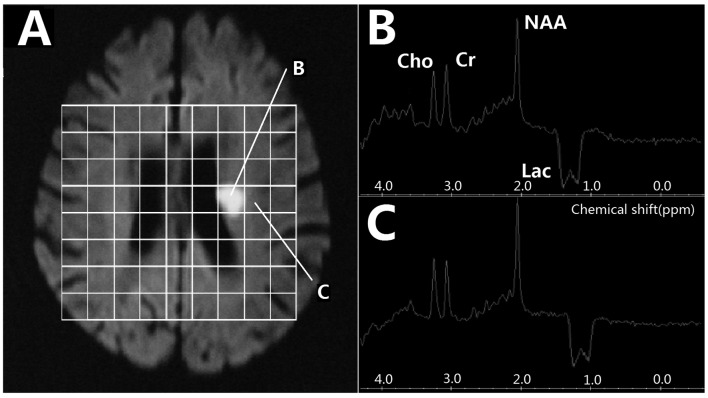
Proton magnetic resonance spectroscopy (^1^H-MRS) in acute stage. (A) MRS map; (B) MRS of infarction center; (C) MRS of border region.

All 72 lesions on ^1^H-MRS images showed a visible inverted Lac peak at 1.3 ppm, which included 52 bimodal and 20 single peaks. At the center of the lesion, NAA/Cho was significantly higher in the <6 h group than in the 6–12 h group, while the Lac/Cr was notably lower in the <6 h group than in the 6–12 h group (t=2.593, P=0.011; t=2.630, P=0.010; t=5.478, P<0.001, respectively). However, in the border region, the NAA/Cr and NAA/Cho ratios were only slightly decreased and an inverted Lac peak was present (Fig. 1 and Table I). Of the 47 patients, 21 (29 lesions) underwent the first ^1^H-MRS examination within 6 h after stroke and 26 (43 lesions) underwent ^1^H-MRS between 6–12 h afterwards. At the center of the lesion, NAA and Lac were decreased in the <6 h group compared with those in the 6–12 h group. However, the differences between the <6 h and 6–12 h groups were not statistically significant at the infarction border region. The comparison of MRS values in the central and border region of lesions at different time points are presented in Table II.

### ^1^H-MRS findings on reexamination

Of the 47 patients, 43 were reexamined and 4 were lost to follow-up. Sixty-nine lesions were identified at the reexaminations and 61 were typical infarction lesions with long T_1_ and T_2_ signals and clear boundaries. Compared with the first DWI results, 45 lesions were enlarged, eight had disappeared, five were reduced and 11 had no marked change. At the center of the lesion, the NAA/Cr and NAA/Cho ratios were decreased and the Lac/Cr value was reduced in the follow-up (1–2 months group) group compared with those in the <12 h group, and the differences were statistically significant (t=7.933, P<0.001; t=3.962, P<0.001; t=6.531, P<0.001, respectively). However, at the infarction border region, the only difference observed between the <12 h and follow-up groups was that the Lac/Cr ratio was decreased (t=9.875, P<0.001). A comparison of the lesion center and border region metabolite values between the cerebral infarction acute stage and 1–2 months after stroke (median follow-up time, 1.4 months; range, 1.1–1.9 months) is presented in Table III. At the infarction center, no statistically significant differences were identified between the NAA and Lac values in the thrombolysis and non-thrombolysis groups. However, at the infarction border region, the NAA/Cr and NAA/Cho values were lower in the non-thrombolysis group compared with those in the thrombolysis group. A comparison of follow-up MRS values between patients with and without thrombolytic treatments is shown in Table IV.

## Discussion

The results of this study indicate the manner in which ^1^H-MRS is able to supplement the better resolution, but less specific morphological findings of MRI, particularly as it provides information concerning the distribution of NAA and Lac as markers of intact neuronal tissue. Compared with single-volume spectroscopy, MRS imaging allows a more precise definition of the center of the infarct since this imaging may be analyzed retrospectively. In addition, ^1^H-MRS may be used noninvasively and *in vivo* to determine the concentrations of intracellular metabolite in the infarcted brain that may predict the tissue viability of the brain structures enclosing the infarcted area.

In this study, 72 lesions were tested by ^1^H-MRS examination. Within 6 h after stroke, it was observed that Lac/Cr ratios in the infarction center and border region decreased compared to the Lac/Cr ratios on the contralateral side, which indicated brain tissue ischemia. In the infarction center, NAA/Cr and NAA/Cho ratios significantly decreased compared with those in the control side, while only a slight reduction of the NAA/Cr and NAA/Cho ratios was observed in the infarction border region, which indicated more serious anoxic damage of cells in the infarction center. Lac is the end product of the anaerobic metabolism of glucose. It is a marker of cell energy metabolism. Under normal circumstances, the metabolism of the brain cell (neuron) is mainly aerobic metabolism, so the level of lactic acid is very low. Lac levels are elevated when the oxygen supply is insufficient, which indicates ischemia in the territory of a blood vessel to the brain (12). Previous studies (13,14) showed that the oxygen consumption rate of normal brain tissue is 20 ml/100 g/min. Lac is generated when the metabolism of cerebral blood flow is below this value. Thus the appearance of Lac is considered to be a sensitive marker in the early stage of infarction and it appears before the routine MRI examination shows abnormal changes. Cho includes glycerol phosphate, choline, phosphorylcholine and phosphatidylcholine. Cho is a marker of the cell membrane and sphingomyelin. Its peak is determined by the concentration of choline existing in membrane phospholipids and of acetylcholine used as a neurotransmitter. Pathophysiological processes cause the decomposition of sphingomyelin and an increase in the number of cells may lead Cho levels to increase. Such processes are ischemia, cancer and brain tumors (15). The Cr value represents the total concentration of creatine and phosphocreatine. Its content is relatively constant and uniform in different metabolic conditions of the human brain, particularly the pathological conditions. Therefore it is often used as an internal standard to measure the levels of other metabolites (16). Our results indicate that Lac is a sensitive marker of anaerobic metabolism following cerebral ischemia, but the neuronal damage is secondary to cerebral ischemia and the reduction of the level of NAA occurred after the level of Lac increased. These results are in accordance with those in previous reports (17,18), in which acute cerebral infarction patients were studied, and it was observed that NAA levels were decreased.

NAA is a marker of neuronal density and activity. In addition, NAA is a specific marker that may reflect infarction-related injury. Lac is a product of anaerobic glycolysis. The increase in size of the Lac peak reflects the lack of oxygen supply and indicates the presence of infarction, but not necessarily the development of irreversible cerebral infarction (19). The current study compared MRS values between 6–12 h with values within 6 h, and the results showed that the Lac/Cr ratio remained increased after 6 h of infarction in the infarction center, but the NAA/Cho ratio was decreased. There were only slight changes of MRS values in the border region and the differences were not statistically significant. This indicated that cerebral infarction has a therapeutic time frame.

The ischemic penumbra is the main therapeutic target in cerebral infarction, and also is the main target of the treatment for the progressive development of acute cerebral infarction. Our study results showed that in the border region, the NAA/Cr, Cho/Cr and NAA/Cho ratios differed between the patients who received thrombolytic therapy and those who did not, but there was no significant difference in the Lac/Cr ratio. By contrast, in the infarction center, the difference in each MRS value between the thrombolysis and non-thrombolysis groups was not statistically significant. This result suggests that the infarction border region is the therapeutic target of cerebral infarction, and patients with indications for thrombolysis should be provided with thrombolytic therapy as soon as possible, and the monitoring of the NAA peak is also an important indicator for assessing the effectiveness of cerebral infarction treatment.

In the current study, a technical factor impacted the multi-voxel ^1^H-MRS information collection from the patients with cerebral infarction. The time span of the study was relatively long and a drift in magnetic field often occurs after magnetic resonance has been conducted for numerous hours. Therefore, we adopted the MRS ratio rather than the absolute value to reduce interference. In addition, the level of Cr reflects the metabolic status of mitochondrial function and cellular energy. Although the majority of studies consider that the content in the body is relatively uniform and constant, the level of Cr often changes slightly in areas of membrane damage, such as in cerebral ischemia (20,21). This may also have certain impacts on the study results.

In conclusion, important information for the diagnosis and treatment of cerebral infarction may be provided by the early neural metabolic changes in cerebral infarction patients. An increased Lac/Cr ratio, as well as decreased NAA/Cr and NAA/Cho ratios often indicate irreversible infarction, but if only the Lac/Cr ratio is increased does this indicate the occurrence of cerebral ischemia. The monitoring of the NAA peak may be considered as an indicator for evaluating the effectiveness of treatment for cerebral infarction. Thrombolytic therapy should be administered as early as possible to patients who meet the thrombolytic indications, since it is beneficial for saving tissue activity in the border region between the infarction and normal tissue. However, MRS examination is demanding due to the high strength of the magnetic resonance field and there are numerous interference factors. In addition, there is no unified standard MRS value for the diagnosis of cerebral infarction. Therefore, further study and improvements are required.

## Figures and Tables

**Table I:** Comparison of metabolites among different areas of lesion and control in the acute stage of cerebral infarction (<12 h).

		Infarction center				Infarction border region			
			
Group	n	NAA/Cr	Cho/Cr	NAA/Cho	Lac/Cr	NAA/Cr	Cho/Cr	NAA/Cho	Lac/Cr
Infarction center	72	1.64±0.41^a^	0.99±0.21	1.65±0.45^a^	−1.34±0.37^b^	1.76±0.59	0.92±0.27	1.91±0.34	−0.76±0.09^b^
Contralateral side	72	1.84±0.51	0.97±0.17	1.89±0.63	−0.08±0.03	1.85±0.47	0.89±0.16	2.07±0.61	−0.15±0.18

a P<0.05 vs. control;

b P<0.001 vs. control.

NAA, N-acetylaspartic acid; Cr, creatine; Cho, choline; Lac, lactate.

**Table II:** Comparison of metabolites in two areas at different time windows in the cerebral infarction acute stage.

		Infarction center				Infarction border region			
			
Group	n	NAA/Cr	Cho/Cr	NAA/Cho	Lac/Cr	NAA/Cr	Cho/Cr	NAA/Cho	Lac/Cr
<6 h	29	1.68±0.29	0.96±0.17	1.75±0.49^a^	−1.29±0.27^a^	1.79±0.24	0.91±0.09	1.97±0.29	−0.73±0.26
6–12 h	43	1.56±0.27	1.03±0.22	1.51±0.38	−1.49±0.35	1.75±0.21	0.93±0.10	1.88±0.26	−0.79±0.19

a P<0.05 vs. 6–12 h group.

NAA, N-acetylaspartic acid; Cr, creatine; Cho, choline; Lac, lactate.

**Table III:** Comparison of metabolites in two areas between the cerebral infarction acute stage (<12 h) and 1–2 months after stroke.

		Infarction center				Infarction border region			
			
Group	n	NAA/Cr	Cho/Cr	NAA/Cho	Lac/Cr	NAA/Cr	Cho/Cr	NAA/Cho	Lac/Cr
<12 h	72	1.64±0.41^a^	0.99±0.21^b^	1.65±0.45^a^	−1.34±0.37^a^	1.76±0.59	0.92±0.27	1.91±0.34	−0.76±0.09^a^
1–2 months	69	0.43±0.12	0.69±0.17	0.62±0.14	−0.07±0.11	1.62±0.43	0.89±0.22	1.82±0.29	0.03±0.01

a P<0.001 vs. 1–2 months;

b P<0.05 vs. 1–2 months.

NAA, N-acetylaspartic acid; Cr, creatine; Cho, choline; Lac, lactate.

**Table IV:** Comparison of metabolites of two areas between the early thrombolysis group and non-thrombolysis group 1–2 months following infarction.

		Infarction center				Infarction border region			
			
Group	n	NAA/Cr	Cho/Cr	NAA/Cho	Lac/Cr	NAA/Cr	Cho/Cr	NAA/Cho	Lac/Cr
Thrombolysis	21	0.52±0.44	0.73±0.37	0.71±0.42	−0.08±0.07	1.77±0.61^a^	0.86±0.22^a^	2.05±0.31^a^	−0.02±0.05
Non-thrombolysis	48	0.34±0.32	0.65±0.29	0.52±0.35	−0.06±0.04	1.39±0.43	0.76±0.13	1.82±0.24	−0.03±0.05

a P<0.05 vs. non-thrombolysis group.

NAA, N-acetylaspartic acid; Cr, creatine; Cho, choline; Lac, lactate.
